# Reclassification of *Eimeria pogonae* Walden (2009) as *Choleoeimeria pogonae comb. nov*. (*Apicomplexa*: *Eimeriidae*)

**DOI:** 10.1007/s00436-015-4787-2

**Published:** 2015-10-14

**Authors:** Klaudiusz Oktawian Szczepaniak, Krzysztof Tomczuk, Anna Lojszczyk-Szczepaniak, Wojciech Lopuszynski

**Affiliations:** Faculty of Veterinary Medicine, University of Life Sciences in Lublin, Akademicka 13, Lublin, 20-950 Lubelskie Poland

**Keywords:** Biliary coccidium, Bearded dragon, Liver, Coccidiosis

## Abstract

The presented paper provides a reclassification of *Eimeria pogonae* from *Pogona vitticeps* into the correct genus *Choleoeimeria*. A description of exogenous and endogenous stages of biliary coccidium is given. Sporulation of the oocysts was endogenous. The mature oocysts contained four sporocysts each with two sporozoites. Oocysts were ellipsoidal in shape, with average length/width ratio 1.7 and measured 28.4 (SD1.5) × 16.8 (SD 1.5). The micropyle, residuum, and polar granules were absent from the sporulated oocysts. Ovoidal in shape, sporosysts without Steida bodies contained residuum and two elongated and boat-shaped sporozoites. The endogenous stages of the coccidia were located mainly in the epithelium of bile ducts; however, single-epithelium cells of the gallbladder were also infected.

## Introduction

One of the most popular lizards kept individually as an exotic pet is the bearded dragon (*Pogona vitticeps*). This is a species native to Central Australia, widely available in world pet trade. Common endoparasites detected in bearded dragons are coccidia (Raiti 2012). To date, only two coccidia parasitizing *P. vitticeps* have been described (McAllister et al. 1995; Walden 2009).

*Isospora amphiboluri* is a parasite with relatively strict host specificity for the Australian agamid lizard from the genus *Pogona*, reported in both wild and captive populations. This species was first described by Cannon (1967), in *Pogona barbata*, and re-described by McAllister et al. (1995) in *P. vitticeps*. Isosporosis of young bearded dragons is usually associated with high mortalities while in adult individuals, *I. amphiboluri* seem to be a parasite of low pathogenicity. In the whole life cycle of the endogenous stage, this parasite appears to be limited to the epithelial cells of the intestine.

Novel *Eimeria* sp. oocysts in faecal samples of *P. vitticeps* obtained from two captive breeding populations were observed by Walden (2009). Approximately 80 % of these oocysts were sporulated in fresh faeces. All oocysts were ellipsoidal with an average size and shape index (*n* = 20) of 27.2 × 15.1 μm and 1.8, respectively. According to the morphological characteristics of the detected oocysts, the author placed this new species within the genus *Eimeria* and proposed the name *Eimeria pogonae* after the host’s generic name. Their description was only limited to the exogenous stages, but nothing was reported about the infection site and endogenous development. However, for a correct taxonomic classification of a reptile coccidian, it is fundamental to follow its host localization and mode of development in the endogenous stages.

Some reptilian coccidia which undergo endogenous development in the gallbladder epithelium, previously described as *Eimeria*, were reclassified by Paperna and Landsberg (1989) as a separate genus referred to as *Choleoeimeria*. The molecular separation of the genera *Eimeria* and *Choleoeimeria* was confirmed by Jirků et al. (2002). Despite the fact that many species of *Choleoeimeria* were recognized in different reptilian families to date (Abdel-Baki 2014; Abdel-Baki et al. 2014; Al-Quraishy et al. 2013), there has been no study on determining the occurrence of *Choleoeimeria* spp. in bearded dragons.

The presented paper provides a morphological and morphometrical characterization of oocyst structures of the coccidians isolated from the gallbladder and intestinal content of *P. vitticeps*. Further, a description is given of localization of endogenous development and histopathological changes in the epithelial cells of the gallbladder. According to the oocyst morphology and infection site, we classified the coccidium as belonging to the genus *Choleoeimeria*.

## Materials and methods

A total of 24 dead adults, naturally infected with coccidian bearded dragons, were delivered to the laboratory of Department of Parasitology and Invasive Diseases, Faculty of Veterinary Medicine, University of Life Sciences, Lublin, Poland. All of the animals came from a commercial farm of captive reptiles. Necropsy examinations and samples collections were done within a few hours of the lizards’ death. Their intestinal contents or faeces (obtained from rectum or/and cloaca) were examined using Sheather’s sugar flotation method. The gallbladder contents were examined in wet mounts. Sporulated oocysts were observed via a light microscope fitted with Nomarski interference-contrast (DIC). For morphometric data, only sporulated oocysts (at least 50 from each individual) were used. Measurements were made from photographs obtained of the living oocysts, without any signs of degeneration, in accordance with guidelines set by Duszynski and Wilber (1997).

To study the endogenous stages, samples of the intestines, liver and gallbladder were fixed in 10 % formalin, pH = 7.2 for 24 h, and routinely processed and embedded in paraffin blocks. The histological sections were stained with haematoxylin and eosin. All measurements were given in micrometres (μm).

## Results

**Fig. 1:**
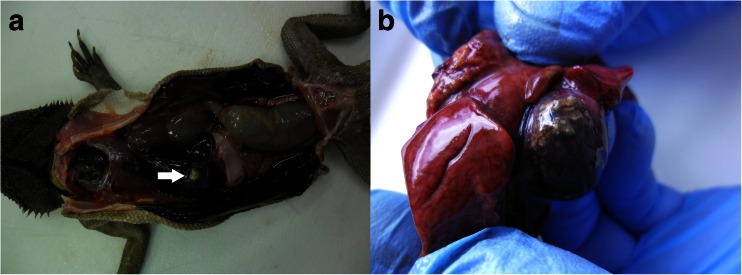
Macroscopically visible pathological changes of the gallbladder in infected lizards. Local white wall thickening (**a**). Gallstones and debris inside the gallbladder (**b**)

Sixteen necropsied animals were in poor nutritional condition with visible signs of emaciation and dehydration, while rest eight were cachexic. A necropsy revealed underdeveloped fat bodies in all lizards. Slight jaundice in the mucous and serous membranes was seen in 13 individuals. Twelve lizards had significant gallbladder dilatation and local, white wall thickening. Inside the gallbladder of 16 lizards were macroscopically visible masses of small gallstones (about 1–3 mm in diameter) and/or debris (Figs. 1a, b).

All the examined animals were coccidia positive. Oocysts were present both in the intestinal contents (11/24) and the bile (24/24). All sporulated oocysts obtained from the intestinal content and bile have the same morphotype and did not differ significantly in size, but the number of oocysts in the bile and intestinal content varied. Numerous oocysts were observed in the gallbladder while only single ones in the intestine. A histological section of the gallbladder demonstrated that most of the oocysts from the bile were aggregated in dense clusters.

Oocysts isolated from the intestine were fully sporulated (four sporocysts with two sporozoites each), while oocysts from the gallbladder were in different stages of development. Forty percent of the observed oocysts were developed to the stadium of four sporocysts per oocyst. In 35 % of the oocysts, sporozoites were released from the sporocysts and eight separate sporozoites were seen inside an oocyst. Only 2 % of the oocysts were unsporulated. One percent of the oocysts, present in bile smears from all the examined lizards, were ruptured, while single, free sporozoites released from the oocysts were observed in the bile. The remaining oocysts (22 %) were empty or contained numerous granuloma structures, but the wall of these oocysts seemed to be unaltered.

**Fig. 2:**
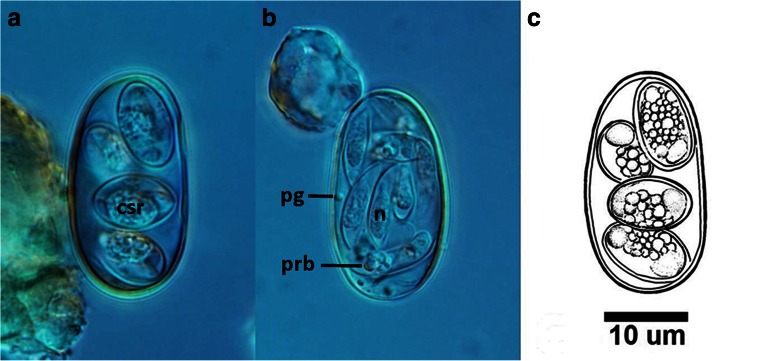
Photographs of ooccyst of *Choleoeimaria pogonae*. **a** Sporulated oocyst containing four sporocysts each with two sporozoites. Note compact sporocyst residuum (*csr*). **b** Sporulated oocyst isolated from the gallbladder with eight sporozoites released from sporocysts. Note polar granule (*pg*), posterior refractile body of the sporozoite (*prb*) and sporozoite nucleus (*n*). **c** Line drawings of sporulated oocysts. All in the same *scale bar* = 10 μm

The mature oocysts were ellipsoidal in shape, measured 28.4 (SD 1.5) × 16.8 (SD 1.5) (25.6–32.3 × 13.6–21.4). The length/width ratio (L/W) was 1.7 (SD 0.1) (1.4–2.2). The wall was double-layered measuring 0.7 (0.6–1.1) in thickness, with a smooth surface texture. The micropyle, residuum and polar granules were absent from the sporulated oocysts. Ovoidal in shape, sporocysts without Steida bodies contained residuum and two sporozoites. The average sporocyst size was 12.5 (SD 1.5) × 6.6 (SD 0.5) (9.4–14.7 × 5.3–7.4); the length/width ratio (L/W) was 1.8 (SD 0.2) (1.4–2.3). On the surface of sporocyst wall, longitudinal suture was seen. Sporozoites were elongated and boat-shaped, slightly tapering at the anterior and posterior ends, possessing a smooth surface, measured 13.3 × 3.5 (12.7–14.1 × 3.2–3.7), with length/width ratio 3.8 (3.7–3.9). Each of them had one distinct posterior refractile body 2.8 (2.5–3.0) (Fig. 2a–c).

**Fig. 3:**
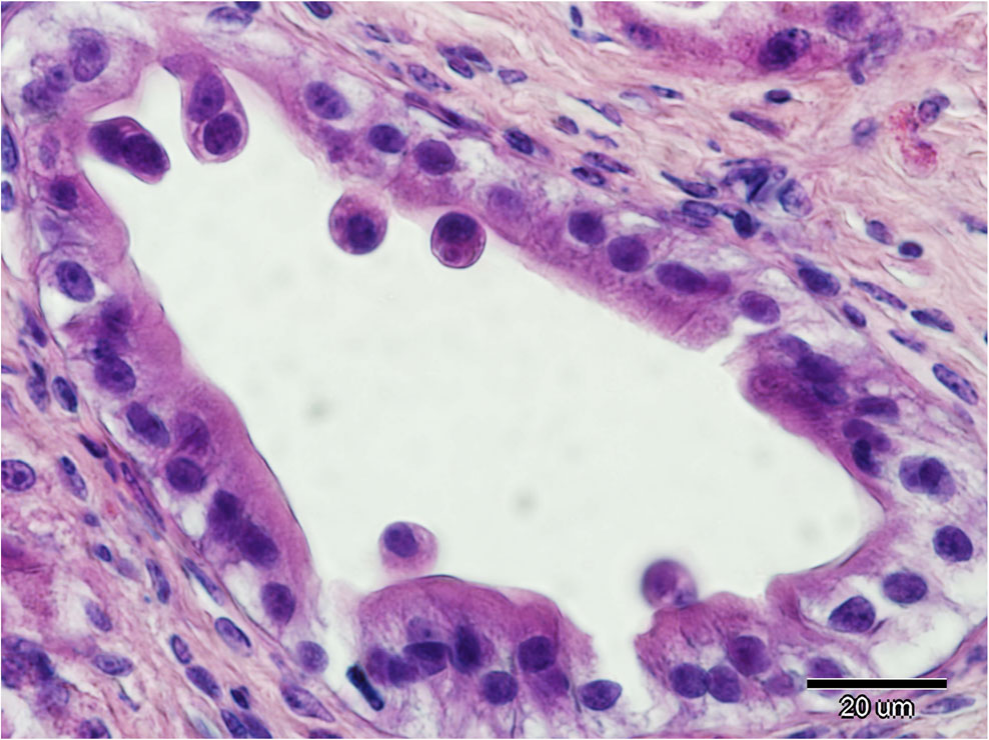
Various endogenous stages of coccidia. Note infected cells are displaced into the lumen of bile duct

The results of the histopathological examination confirmed endogenous development of coccidia in the epithelium of the gallbladder and bile ducts. Numerous endogenous stages of coccidia were seen in the epithelial cells of the medium and large bile ducts, while only single host cells were infected in the gallbladder. A microscopic analysis revealed various endogenous stages of coccidia (Figs. 3). The infected cells were displaced into the lumen of bile duct or gallbladder and attached to the basement membrane through a stalk of cytoplasm. In many regions of the gallbladder, mucosal abnormalities such as focal pseudostratification, epithelial hypertrophy and cell degeneration were present. The length of uninfected cells of gallbladder epithelium differed significantly, ranging from 10.30 to 44.20 μm. In the subepithelial connective tissue, numerous heterophils were observed.

No endogenous stages of the coccidia were detected in intestinal samples.

## Discussion

Traditional taxonomy of coccidian parasites invading reptiles placed all species together with oocysts that have four dizoic sporocysts into the genus *Eimeria* (Jirků et al. 2009; Levine 1985). This classification was based primarily on the morphology of the exogenous stages (oocysts, sporocysts, sporozoites), while less focused on information about the life cycles and biology of these coccidia. Therefore, recent studies on endogenous development, sporocyst excystation structures as well as molecular analysis all lead to a reclassification of some reptilian coccidia (Paperna 2007). In fact, all tetrasporic dizoic coccidia parasitizing reptilian hosts have been split into three genera: *Eimeria*, *Acroeimeria* and *Choleoeimeria* (Tenter et al. 2002; Lainson and Paperna 1999).

Members of *Choleoeimeria* have a specific location restricted to the gallbladder epithelium of reptiles, mainly lizards, and less commonly to snakes and tortoises. Usually, the epicytoplasmatic development of the endogenous stages leads to hypertrophy of gallbladder epithelium and displacement of host cells into the bile lumen. Mature oocysts contain four sporocysts each witch two sporozoites. Oocysts are characterized by a lack of micropyle, oocyst residuum, and are generally uniform with a typical cylindroidal or ellipsoidal shape (length/width ratio 1.6–2.2). Sporocysts have neither the Stieda nor substieda bodies (Jirků et al. 2009; Sloboda and Modrý 2006; Tenter et al. 2002). Another important feature for the classification of the coccidia is excystation structures (Box et al. 1980). In *Choleoeimeria*, the sporocyst wall is composed of two valves joined by a longitudinal suture (Jirků et al. 2002; Paperna 2007).

Based on the results of morphological analysis of mature oocysts and the location of the endogenous stages, we conclude that the invasion observed in the present study was caused by the genus *Choleoeimeria*. According to literature, all species placed within *Choleoeimeria* are homoxenous and strictly host specific (Jirků et al. 2009). Therefore, we compared our results only with Eimeria-like coccidia parasitizing the Australian agamid from the genus *Pogona*. Up to now, no species of *Choleoeimeria* have been reported in any of the eight species that belong to the genus *Pogona* (Cannon 1967; McAllister et al. 1995; Walden 2009). Only one tetrasporocystic dizoic coccidian had been previously described in *P. vitticeps*. Based solely on the morphology of the exogenous stages, Walden (2009) placed them in the genus *Eimeria* and classified as *E. pogonae*, despite the fact that the oocysts described by him had features typical of genus *Choleoeimeria*. Although Walden (2009) suspected that the location of the endogenous stages for *E. pogonae* was in the epithelium of the gallbladder, he did not determine the site of infection. Unfortunately, Walden’s Ph.D. thesis was not published in scientific revived journal, but his results should not be ignored; however, taxonomic revision of *E. pogonae* is needed. In fact, the oocysts of *E. pogonae* are indistinguishable in morphology from those found by the present study, which indicate that these two coccidians belong to the same genus—*Choleoeimeria*. Due to a generally narrow host specificity of *Choleoeimeria* spp., the chances that the coccidian in the present study is different from that of Walden study are extremely low. Therefore, most probably, both coccidians are conspecific. Considering morphological and developmental traits, we propose reclassification of *E. pogonae* and transfer it into the correct genus as *Choleoeimeria pogonae comb. nov*.

Small differences in the size of oocysts and sporocysts in both studies might be related to use of different microscopic optic or can be accounted for duration of infection. Duszynski (1971) found that oocysts appearing the earliest in infection were smaller than those observed later during patency, whereas the shape index remained more or less constant. In the present study, all of the examined bearded dragons were in late stages of infection, which was confirmed by the results of the autopsy and presence of sporulated oocysts in the gallbladder. In general, sporulation of oocysts in the genus *Choleoeimeria* is endogenous. However, according to Sloboda and Modrý (2006), localization of this process is time dependent and may occur in intestine or gallbladder as well as outside the host. In the initial phase of the patent period, all oocysts observed in faeces are unsporulated, but the percentage of fully sporulated oocysts increases during patency. In later stages of infection, most oocysts probably remain in the gallbladder, having enough time to sporulate. In our study, the majority of oocysts recovered from the gallbladder were fully sporulated and only 2 % remained unsporulated, while in Walden’s study, 20 % of oocysts isolated from fresh faeces samples were unsporulated, which suggests an early stage of infection.

In heavily infected animals, oocyst shedding can be totally stopped by obturation of the bile ducts caused by pathological hypertrophy of epithelial cells and tissue debris (Sloboda and Modrý 2006). In the present study, only single-epithelial cells in gallbladder were infected with endogenous stages of coccidian, but most of them were hypertrophied. There is limited information on the pathology of reptilian gallbladder; however, the pathological hypertrophy of the mucosa of biliary vesicle is frequently identified as the characteristic response for the presence of gallstones in mammalian (Mathur et al. 2012). It is possible that in dissected animals, most of the epithelial abnormalities in gallbladders were caused by chronic inflammation induced by conglomerates of oocysts accompanied with precipitates of tissue debris. Our results of the histopathological examination confirmed this thesis. The largest hypertrophy of the gallbladder’s mucosa was observed in 16 lizards with macroscopically visible gallstones or debris.

All these factors may lead to the lack of oocysts in the faecal samples in late patent period and some difficulties in diagnosis of reptile choleoeimeriosis using standard coprological methods. Our observations indicate low sensitivity of faecal flotation. Indeed, in 13 of 24 dissected animals, we observed numerous oocysts only in their gallbladders, while results of parasitological investigations of faeces or intestinal contents were negative. A similar proportion of false-negative results was determined by Sloboda and Modrý (2006) in a faecal examination of *Chamaleo calyptratus*. The consequence of such false-negative results of faecal examination is probably an underestimation of the prevalence of *Choleoeimeria* spp. invasions in captive and wild reptiles.

On the other hand, using coprological diagnostic methods for detection of *Choleoeimeria* sp. in early stage of infection can be also problematic, due to relatively long prepatent period. As estimated by Sloboda and Modrý (2006) in their experimental trials, oocysts of *Choleoeimeria hirbayah* were evacuated with the faeces within 50–83 days of infection. Several authors also suggest that in early stages of patent period (when oocysts are present in faeces) animals usually demonstrate good condition and nutrition status (Abdel-Baki 2014; Al-Quraishy et al. 2013; Lainson and Paperna 1999). Because routine parasitological examinations of asymptomatic reptiles are not a common veterinary practice, most animals are simply not diagnosed. This fact may explain why in *P. vitticeps*—one of the most common lizards in individual breeding—invasion of the genus *Choleoeimeria* has not been described so far. In contrast to invasion of *I. amphiboluri*, which is usually associated with clinical signs such as diarrhea and progressive apathy in captive bearded dragons (Raiti 2012). The pathogenicity potential of *C. pogonae* is not clear for us. To establish accurate disease state associated with biliary coccidiosis in bearded dragons of different ages and different stages of invasion, clinical and pathological data conducted in experimental trials are necessary.
